# Power Efficient Secure Full-Duplex SWIPT Using NOMA and D2D with Imperfect CSI

**DOI:** 10.3390/s20185395

**Published:** 2020-09-21

**Authors:** Jingpu Wang, Xin Song, Yatao Ma, Zhigang Xie

**Affiliations:** College of Computer Science and Engineering, Northeastern University, Shenyang 116026, China; 1610533@stu.neu.edu.cn (J.W.); 1871644@stu.neu.edu.cn (Y.M.); 1710566@stu.neu.edu.cn (Z.X.)

**Keywords:** multi-objective optimization (MOO), secure full-duplex simultaneous wireless information and power transfer (FD-SWIPT), non-orthogonal multiple access (NOMA), device-to-device (D2D), imperfect channel state information (CSI)

## Abstract

The secure full-duplex (FD) simultaneous wireless information and power transfer (SWIPT) system and non-orthogonal multiple access (NOMA) have been deemed two promising technologies for the next generation of wireless communication. In this paper, the network is combined with device-to-device (D2D) and a practical bounded channel state information (CSI) estimation scheme. A system total transmit power minimization problem is studied and formulated as a multi-objective optimization (MOO) problem via the weighted Tchebycheff approach. A set of linear matrix inequalities (LMI) is used to transform the non-convex form of constraints into the convex form. Considering the imperfect CSI of the potential eavesdropper for robust power allocation, a bounded transmission beamforming vector design along with artificial noise (AN) is used, while satisfying the requirements from the secrecy rates as well as the energy harvesting (EH) task. Numerical simulation results validate the convergence performance and the trade-off between the uplink (UL) and downlink (DL) data transmit power. It is also shown that by FD and NOMA, the performance of the proposed algorithm is higher than that of half-duplex (HD) and orthogonal multiple access (OMA).

## 1. Introduction

Next-generation communication systems require self-sustainability wireless nodes to maintain a high data rates network and guarantee quality of service (QoS) [1]. Radio frequency (RF) signal-based simultaneous wireless information and power transfer (SWIPT) is a promising technique for prolonging the lifetime of continuous network operation [2,3]. SWIPT can jointly extract information and replenish energy from the same signal by performing two circuits to separate the information processing and power transfer alternatively [4,5]. Non-orthogonal multiple access (NOMA) has also become a key issue of the novel energy and spectrum efficient technologies due to a higher network capacity compared with orthogonal multiple access (OMA) in the next-generation communication networks [6]. NOMA can provide the same resource (e.g., time/frequency/code) for multiple users by using different a power level in one subcarrier [7,8]. The combination of NOMA and device-to-device (D2D) communication is essential for alleviating the traffic burden on future networks. In contrast to the traditional concept of “D2D pair”, the concept of “D2D group” involves several D2D receivers that are capable of receiving information from a single D2D transmitter. To further improve the system spectrum efficiency (SE), the full-duplex (FD) transceiver is considered as it can be adopted in simultaneous downlink (DL) and uplink (UL) transmission in the same frequency band [9]. However, FD NOMA communication is more susceptible to eavesdropping compared to conventional half-duplex (HD) OMA. Further, this situation also causes extra energy consumption in SWIPT.

In practical systems, secrecy is a critical concern for the design of wireless communication protocols due to the broadcast nature of the wireless medium [10]. Physical security techniques can improve secure wireless information transmission by generating more interference to potential eavesdroppers [11]. By adding artificial noise (AN) and projecting it onto the null space of information user channels in information transmit beamforming, the potential eavesdroppers would experience a higher noise floor and thus obtain less information about the messages transmitted to the legitimate receivers [12]. In SWIPT systems, the secure communication problem is severer because of larger power consumption in the energy harvesting (EH) task [13]. Besides, taking into account that the base station (BS) may not perfectly know the channel state information (CSI) of the roaming users (RU) also creates a potential vulnerability of ensuring secure communications [10]. 

Most of the studies mentioned above rely on one case that the BS can get the perfect knowledge of CSI. However, in practice, the BS always has imperfect CSI. To deal with it, we assume a channel estimation error model where the BS only knows the estimated channel gain and a prior knowledge of the variance of the estimation error [14]. In this paper, we consider D2D-aided FD NOMA-enhanced secure SWIPT communications with imperfect CSI, in which D2D receivers can reuse the same subcarrier occupied by the information transmit user to improve the spectrum utilization in the power domain NOMA. To the best of our knowledge, the existing works cannot use a power efficiency algorithm in secure NOMA- and D2D-enhanced FD SWIPT systems with channel estimation and energy constraints. To study the problem, we propose an algorithm that involves constraints from EH and secure information transmission tasks to jointly extract information and replenish energy. Hence, the proposed algorithm needs to transform the probabilistic non-convex optimization problem into a bounded convex optimization problem. Besides, the existence of a trade-off between UL and DL co-channel interference (CCI) in the FD system needs to be solved by a multi-objective optimization method. Considering all the sub-problems above, a Pareto optimal policy is able to be defined with semidefinite programming (SDP) relaxation [15]. After that, we can iteratively minimize the bounded power allocation coefficients for the objective function of the optimization problem by CVX and guarantee security and QoS, simultaneously. 

The rest of the article is organized as follows. The channel model and problem formulation are introduced in Section 2. Then, the proposed algorithm is elaborated in Section 3. In Section 4, we talk about the simulation results, while Section 5 finally draws the conclusions of this work.

Notation:$A^{-1},A^{H},\mathrm{Tr}\left(A\right),\mathrm{Rank}\left(A\right)$ and det(A) denote the inverse, Hermitian transpose, trace, rank and determinant of matrix A, respectively; $\mathrm{diag}\left(x_{1},...,x_{M}\right)$ is a diagonal matrix with the diagonal elements given by $\left\{x_{1},...,x_{M}\right\}$, and diag(A) returns a diagonal matrix having the main diagonal elements of A on its main diagonal; ${\left[x\right]}^{+}$ stands for max{0,x} [16]. In addition, the abbreviations in this work are summarized in Table 1.

## 2. Network Model and Problem Formulation 

In this section, the considered FD NOMA and D2D network in SWIPT along with the channel models is presented. Further, in the formulation of this problem, we first define the secure transmission problem in SWIPT employing a resource allocation scheme and give the imperfect CSI channel model. Then, a non-convex optimization problem is presented with the resource allocation design. 

### 2.1. Network Model

**Figure 1:**
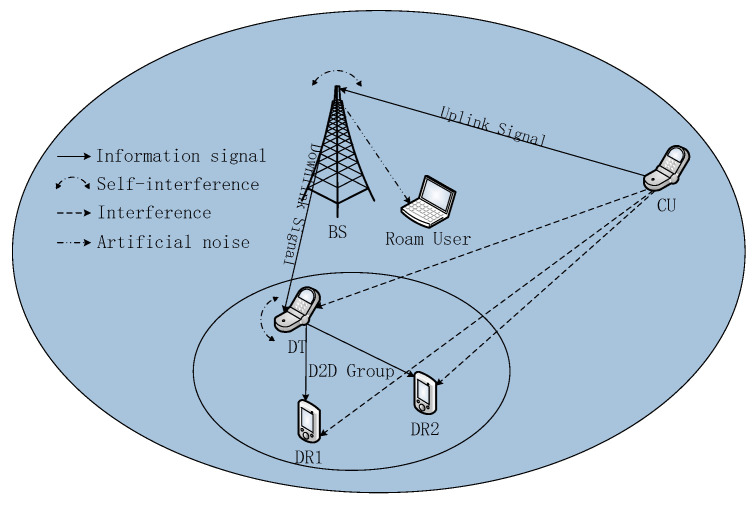
Full-duplex (FD) non-orthogonal multiple access (NOMA)-based simultaneous wireless information and power transfer (SWIPT) with the device-to-device (D2D) communications network model with one multiple-antennas base station (BS), four half-duplex (HD) single-antenna trusted users and one HD untrusted user.

We focus on a secure NOMA-based SWIPT UL and DL scenario in a heterogeneous network, as shown in Figure 1, which requires a D2D group communication including one D2D transmitter (DT) and two D2D receivers (DR). In Figure 1, the FD BS is assumed to be equipped with M antennas to facilitate secure transmission. UL cellular users (CU) and three D2D users (DU) are trusted users. Among them, DT in the D2D group is assumed to work in full-duplex mode and is equipped with three antennas. The other trusted users are assumed to have a single antenna for low hardware complexity. All antennas in the user devices are assumed to work in half-duplex (HD) mode. The D2D communication has two missions to accomplish. On each subcarrier, the FD BS transmits a signal to DT and receives a signal from CU. In the same scheduling time slot, two DRs in the D2D group receive two independent signal streams simultaneously from DT in the NOMA way. Each DR uses a successive interference canceller to detect its own signal. Since the network model has an untrusted user with the energy harvesting task, it is treated as a potential eavesdropper with N antennas (N<M). Thus, in the power allocation scheme, we take the untrusted user into account to guarantee secure information transmission of the wireless network.

According to the system model in Figure 1, the signals received by the network devices can be written as follows. The received signal at the FD BS can be written as

(1) $$y_{BS}=\sqrt{P_{UL}}g_{UL}x_{UL}+\underset{\mathrm{self}-\mathrm{interference}}{\underbrace{H_{SI}^{}w_{DT}^{}x_{DT}^{}}}+\underset{\mathrm{artificial}\mathrm{noise}}{\underbrace{H_{SI}^{}z}}+n_{BS}^{}$$

where $P_{UL}$ is the data transmission power from the uplink CU to the FD BS; $g_{UL}\in {\mathbb{C}}^{M\times 1}$ is the uplink channel vector of the CU; ℂ represents the complex matrix; $x_{UL}\in \mathbb{C}$ is the information bearing signal for CU. It is assumed that $E\left[{\left|x\right|}^{2}\right]=1$; $H_{SI}^{}\in {\mathbb{C}}^{M\times M}$ is the self-interference (SI) channel matrix of the FD BS; $w_{DT}\in {\mathbb{C}}^{M\times 1}$ is the corresponding beamforming vector; $x_{DT}$ is the data from the FD BS to DT; z is distributed as a complex Gaussian random vector $N_{C}\left(0,Z\right)$ with mean 0, and represents the AN sent by the FD BS to degrade the signal to noise ratio (SNR) of the eavesdropper or the RU; Z⪰ **0** is the covariance matrix of z and Z∈ **ℋ***^M^*, where **ℋ***^M^* is an M×M Hermitian matrix; and $n_{BS}^{}∼N_{C}\left(0,\sigma^{2}I_{M}\right)$ is the additive white Gaussian Noise (AWGN) from the uplink channel to the FD BS, where $I_{M}$ denotes the M×M identity matrix [14].

The received signal at the DT can be written as

(2) $$\begin{array}{l} y_{DT}=h_{DT}^{H}w_{DT}^{}x_{DT}^{}+\underset{\mathrm{self}-\mathrm{interference}}{\underbrace{H_{DT}^{}\left(w_{DR1}^{}x_{DR1}^{}+w_{DR2}^{}x_{DR2}^{}\right)}}\\ +\underset{\mathrm{artificial}\mathrm{noise}}{\underbrace{h_{DT}^{H}z}}+\underset{\mathrm{UL}-\mathrm{to}-\mathrm{DL}\mathrm{co}-\mathrm{channel}\mathrm{interference}}{\underbrace{\sqrt{P_{UL}}f_{DT}x_{UL}}}+n_{DT}^{} \end{array}$$

where $h_{DT}\in {\mathbb{C}}^{M\times 3}$ is the downlink channel between DT and the FD BS; $H_{DT}\in {\mathbb{C}}^{3\times 3}$ is the SI channel matrix of DT; $\left\{w_{DR1},w_{DR2}\right\}\in {\mathbb{C}}^{3\times 1}$ denote the corresponding beamforming vectors of D2D receivers; $x_{DR1}$ and $x_{DR2}$ are the data from DT to DR; $f_{DT}\in \mathbb{C}$ is the channel between DT and the UL CU; and $n_{DT}^{}∼N_{C}\left(0,\sigma^{2}I_{3}\right)$ is the AWGN from the FD BS to DT, where $I_{3}$ denotes the 3×3 identity matrix.

To study the signals of D2D receivers, we assume that the channel state of DR1 is better than DR2 as DR1 is located far away from the CU and the eavesdropper in Figure 1, and ${\left\|w_{DR1}^{}\right\|}^{2}<{\left\|w_{DR2}^{}\right\|}^{2}.$ In NOMA, DR1 first decodes the signal of DR2 by successive interference cancellation (SIC). The received information signal at DR1 is derived as

(3) $$\begin{array}{ll} y_{DR1}= & h_{DR1}^{H}\left(w_{DR1}^{}x_{DR1}^{}+w_{DR2}^{}x_{DR2}^{}\right)+\underset{\mathrm{multi}-\mathrm{user}\mathrm{interference}}{\underbrace{h_{BS1}^{H}\left(w_{DT}^{}x_{DT}^{}+z\right)}}\\ & +\underset{\mathrm{UL}-\mathrm{to}-\mathrm{DL}\mathrm{co}-\mathrm{channel}\mathrm{interference}}{\underbrace{\sqrt{P_{UL}}f_{DT}x_{UL}}}+n_{DR1} \end{array}$$

where $h_{DR1}\in {\mathbb{C}}^{3\times 1}$ is the downlink channel between DT and DR1; $h_{BS1}\in {\mathbb{C}}^{M\times 1}$ is the downlink channel between FD BS and DR1; $f_{DR1}\in \mathbb{C}$ is the channel between DR1 and the UL CU; and $n_{DR1}∼N_{C}\left(0,\sigma^{2}\right)$ is the AWGN of DR1.

Similarly, the received signal at DR2 can be written as

(4) $$\begin{array}{ll} y_{DR2}= & h_{DR2}^{H}\left(w_{DR1}^{}x_{DR1}^{}+w_{DR2}^{}x_{DR2}^{}\right)+\underset{\mathrm{multi}-\mathrm{user}\mathrm{interference}}{\underbrace{h_{BS2}^{H}\left(w_{DT}^{}x_{DT}^{}+z\right)}}\\ & +\underset{\mathrm{UL}-\mathrm{to}-\mathrm{DL}\mathrm{co}-\mathrm{channel}\mathrm{interference}}{\underbrace{\sqrt{P_{UL}}f_{DT}x_{UL}}}+n_{DR2} \end{array}$$

where $h_{DR2}\in {\mathbb{C}}^{3\times 1}$ is the downlink channel between DT and DR2; $h_{BS2}\in {\mathbb{C}}^{M\times 1}$ is the downlink channel between FD BS and DR2; $f_{DR2}\in \mathbb{C}$ is the channel between DR2 and the UL CU; and $n_{DR2}∼N_{C}\left(0,\sigma^{2}\right)$ is the AWGN of DR2.

As for the potential eavesdropper, here we use RU for convenience, with multiple antennas, and the received signal can be written as

(5) $$\begin{array}{ll} y_{E}= & L_{E}^{H}w_{DT}^{}x_{DT}^{}+\underset{\mathrm{multi}-\mathrm{user}\mathrm{interference}}{\underbrace{L_{DT}^{H}\left(w_{DR1}^{}x_{DR1}^{}+w_{DR2}^{}x_{DR2}^{}\right)}}\\ & +\underset{\mathrm{UL}-\mathrm{to}-\mathrm{DL}\mathrm{co}-\mathrm{channel}\mathrm{interference}}{\underbrace{\sqrt{P_{UL}}e_{E}x_{UL}}}+\underset{\mathrm{artificial}\mathrm{noise}}{\underbrace{L_{E}^{H}z}}+n_{E}^{} \end{array}$$

where $L_{E}\in {\mathbb{C}}^{M\times N}$ is the channel between the RU and the FD BS; $L_{DT}\in {\mathbb{C}}^{3\times N}$ is the channel between RU and DT; $e_{DT}\in {\mathbb{C}}^{N\times 1}$ is the channel between RU and the uplink CU; and $n_{E}∼N_{C}\left(0,\sigma^{2}I_{N}\right)$ is the AWGN of RU, where $I_{N}$ denotes the N×N identity matrix.

### 2.2. Problem Formulation

In this part, we first analyze the achievable rate and secrecy rate for the considered heterogeneous network. Then, we talk about the CSI knowledge in the communication system for the FD BS to control the power resource. In the end, a joint multi-objective UL and DL power minimization problem is formulated for resource allocation.

The achievable rate of CU can be written as

(6) $$r_{CU}=\log_{2}\left(1+\gamma^{UL}\right),$$

With

(7) $$\gamma^{UL}=\frac{P_{UL}{\left|g_{UL}^{H}v_{UL}\right|}^{2}}{\mathrm{Tr}\left(\rho V_{UL}\mathrm{diag}\left(H_{SI}^{}\left(w_{DT}^{}w_{DT}^{H}+z\right)H_{SI}^{H}\right)\right)+\sigma^{2}{\left\|v_{UL}\right\|}^{2}}$$

where $\gamma^{UL}$ denotes the receiving signal-to-interference-plus-noise ratio (SINR) for CU at the FD BS; $v_{UL}\in {\mathbb{C}}^{M\times 1}$ denotes the receiving beamforming vector of FD BS to decode the information from the CU; and $V_{UL}=v_{UL}v_{UL}^{H}$. The CSI on the uplink channel of CU is estimated by zero-force beamforming (ZF-BF). It is adopted because of the computationally efficient performance for resource allocation. Further, its performance is close to the minimum mean square error beamforming (MMSE-BF) when the receiving SINR at the BS is high. Hence, in ZF-BF,$v_{UL}=g_{UL}/\left\|g_{UL}\right\|$ and $\left\|v_{UL}\right\|$ represents the Euclidean vector norm of $v_{UL}$. The residual SI coefficient ρ represents the ratio of the power received at an antenna that reflects the performance of the SI cancellation, here 0<ρ≪1 [15].

The achievable rate of DT can be written as

(8) $$r_{DT}=\log_{2}\left(1+\gamma^{DT}\right),$$

With

(9) $$\gamma^{DT}={\left\|h_{DT}^{H}w_{DT}^{}\right\|}^{2}/\left(\mathrm{Tr}\left(H_{DT}^{}\left(w_{DR1}^{}w_{DR1}^{H}+w_{DR2}^{}w_{DR2}^{H}\right)H_{DT}^{H}\right)+P_{UL}{\left|f_{DT}\right|}^{2}+\mathrm{Tr}\left(h_{DT}^{}h_{DT}^{H}Z\right)+\sigma^{2}\right).$$

where $\gamma^{DT}$ denotes the receiving SINR from the FD BS to DT.

After SIC, the achievable data rate of DR1 is

(10) $$r_{DR1}=\log_{2}\left(1+\gamma^{DR1}\right),$$

with

(11) $$\gamma^{DR1}=\frac{{\left|h_{DR1}^{H}w_{DR1}^{}\right|}^{2}}{{\left|h_{BS1}^{H}w_{DT}^{}\right|}^{2}+P_{UL}{\left|f_{DR1}\right|}^{2}+\mathrm{Tr}\left(h_{BS1}^{}h_{BS1}^{H}Z\right)+\sigma^{2}}$$

where $\gamma^{DR1}$ is the receiving SINR from the DT to DR1.

The achievable rate of DR2 can be written as

(12) $$r_{DR2}=\log_{2}\left(1+\gamma^{DR2}\right),$$

with

(13) $$\gamma^{DR2}={\left|h_{DR2}^{H}w_{DR2}^{}\right|}^{2}/\left({\left|h_{DR2}^{H}w_{DR1}^{}\right|}^{2}+{\left|h_{BS2}^{H}w_{DT}^{}\right|}^{2}+P_{UL}{\left|f_{DR2}\right|}^{2}+\mathrm{Tr}\left(h_{BS2}^{}h_{BS2}^{H}Z\right)+\sigma^{2}\right).$$

where $\gamma^{DR2}$ is the receive SINR from the DT to DR2.

As outlined before, the RU who has the EH task can be treated as a potential eavesdropper to eavesdrop the information signals of UL and DL trusted CU. Hence, to guarantee communication security, the system is designed to have the ability to deal with the circumstance that RU is able to cancel all multiuser interference. Further, to study the system security, we define the channel capacity for the RU. The channel capacity for RU to eavesdrop information of the UL CU can be written as

(14) $$C_{CU-E}=\log_{2}\det\left(I_{N}+P_{UL}X_{E}^{-1}e_{E}e_{E}^{H}\right)$$

where $X_{E}=L_{E}^{H}ZL_{E}+\sigma^{2}I_{N}$ is the interference-plus-noise covariance matrix of RU. 

The channel capacity for RU to eavesdrop information of the DL DT can be written as

(15) $$C_{DT-E}=\log_{2}\det\left(I_{N}+X_{E}^{-1}L_{E}^{H}w_{DT}w_{DT}^{H}L_{E}^{}\right)$$

The channel capacity for RU to eavesdrop information of the DL DRs can be written as

(16) $$\begin{array}{ll} C_{DR-E}= & \log_{2}\det(I_{N}\\ & +X_{E}^{-1}L_{DT}^{H}\left(w_{DR1}^{}w_{DR1}^{H}+w_{DR2}^{}w_{DR2}^{H}\right)L_{DT}^{}). \end{array}$$

The achievable secrecy rates of the trusted links in the network are given by

(17) $$r_{CU-\mathrm{Sec}}={\left[r_{CU}-C_{CU-E}\right]}^{+}$$

(18) $$r_{DT-\mathrm{Sec}}={\left[r_{DT}-C_{DT-E}\right]}^{+}$$

(19) $$r_{DR-\mathrm{Sec}}={\left[r_{DR1}+r_{DR2}-C_{CU-E}\right]}^{+}$$

respectively.

Considering the EH task, the total amount of energy harvested by RU can be written as

(20) $$E_{RU}^{}=\mu (\mathrm{Tr}\left(L_{E}^{}L_{E}^{H}Z\right)+{\left\|L_{E}^{H}w_{DT}\right\|}^{2}).$$

where 0≤μ<1 is the energy conversion coefficient of RU. 

Assuming all channels vary slowly in each scheduled time slot, the BS can perfectly get the CSI from the trusted users via handshaking. However, the RU only exchanges the pilot information with the BS in the beginning of the working phase for the EH task. When the location of the wireless network users is changing, the FD BS needs to estimate the CSI of RU to guarantee communication security because the RU can become a potential eavesdropper with channel uncertainty [16]. The channel between FD BS and RU $L_{E}$ and the channel between DT and RU $L_{DT}$, along with the channel between RU and CU $e_{E}$, can be modeled as

(21) $$L_{E}={\hat{L}}_{E}+\Delta L_{E}$$

(22) $$\Omega_{E}≜\left\{L_{E}:{\left\|\Delta L_{E}\right\|}_{F}\leq \varepsilon_{E}\right\}$$

(23) $$L_{DT}={\hat{L}}_{DT}+\Delta L_{DT}$$

(24) $$\Omega_{DT}≜\left\{L_{DT}:{\left\|\Delta L_{DT}\right\|}_{F}\leq \varepsilon_{DT}\right\}$$

(25) $$e_{E}={\hat{e}}_{E}+\Delta e_{E}$$

(26) $$\Omega_{e}≜\left\{e_{E}:\left\|\Delta e_{E}\right\|\leq \varepsilon_{e}\right\}$$

where ${\hat{L}}_{E}$, ${\hat{L}}_{DT}$ and ${\hat{e}}_{E}$ are defined to represent the estimated CSI; $\Delta L_{E}$, $\Delta L_{DT}$ and $\Delta e_{E}$ represent the respective channel uncertainties. The continuous sets $\Omega_{E}$, $\Omega_{DT}$ and $\Omega_{e}$ are defined to contain all the possible channel uncertainties with bounded magnitudes $\varepsilon_{E}$, $\varepsilon_{DT}$ and $\varepsilon_{e}$. ${\left\|\cdot \right\|}_{F}$, here, represents the Frobenius matrix norm of $\Delta L_{E}$ and $\Delta L_{DT}$.

Having the CSI, we focus on the transmit power minimization for the system which is an essential issue for green communication. 

Problem 1 (Transmit Power Minimization for the FD BS):

(27) $$\begin{array}{l} \underset{Z,w_{DT},w_{DR1},w_{DR2},P_{UL}}{\mathrm{minimize}}{\left\|w_{DT}\right\|}^{2}+\mathrm{Tr}\left(Z\right),\\ \;s.t.C1:\gamma^{UL}\geq \Gamma_{req}^{UL},\\ C2:\gamma^{DT}\geq \Gamma_{req}^{DT},\\ C3:\gamma^{DR1}\geq \Gamma_{req}^{DR1},\gamma^{DR2}\geq \Gamma_{req}^{DR2},\\ C4:\underset{\begin{array}{l} \Delta L_{E}\in \Omega_{E}\\ \Delta e_{E}\in \Omega_{e} \end{array}}{\max}C_{CU-E}\leq R_{tol}^{CU},\\ C5:\underset{\Delta L_{E}\in \Omega_{E}}{\max}C_{DT-E}\leq R_{tol}^{DT},\\ C6:\underset{\begin{array}{l} \Delta L_{E}\in \Omega_{E}\\ \Delta L_{DT}\in \Omega_{DT} \end{array}}{\max}C_{DR-E}\leq R_{tol}^{DR},\\ C7:E_{RU}^{}\geq P_{\min},\\ C8:P_{UL}\geq 0, \end{array}\;C9:\mathrm{Rank}\left(w_{DT}\right)=1,\mathrm{Rank}\left(w_{DR1}\right)=1,\mathrm{Rank}\left(w_{DR2}\right)=1,\;C10:Z⪰0,w_{DT}≻0,w_{DR1}≻0,w_{DR2}≻0$$

Formula (27) depicts the power allocation optimization problem of minimizing the transmit energy for DT and AN. C1–C3 are the minimum SINR thresholds of the trusted users of the system with $\Gamma_{req}^{\left(\cdot \right)}>0$. Constraints C4–C6 are proposed to guarantee network security. $R_{tol}^{\left(\cdot \right)}$ is the maximum tolerable or the minimum achievable data rate for RU to decode information from the signals of the trusted users in the wireless network. C7 ensures RU gets the minimum required amount of energy. Constraint C8 denotes that $P_{UL}$ is the non-negative power. C9 is a rank-one constraint of $w_{DT}$, $w_{DR1}$ and $w_{DR2}$ to obtain rank-one beamforming. C10 is the constraint of the Hermitian positive semidefinite matrix Z, $w_{DT}$, $w_{DR1}$ and $w_{DR2}$.

Note that the objective function in Problem 1 minimizes the transmit energy from the BS without considering the transmit power from DT and UL CU. Thus, the second network objective function is proposed to minimize the transmit power from DT to DR and total transmit power of UL CU.

Problem 2 (Transmit Power Minimization for DT and CU):

(28) $$\begin{array}{l} \underset{Z,w_{DT},w_{DR1},w_{DR2},P_{UL}}{\mathrm{minimize}}{\left\|w_{DR1}\right\|}^{2}+{\left\|w_{DR2}\right\|}^{2}+P_{UL},\\ s.t.C1-C10. \end{array}$$

Problem 2 targets the transmit power minimization of DT and CU under C1–C10 without considering the power consumed by the FD BS.

From the two sub-problems, we find a trade-off between the two target functions and the existence of the multiple-antennas RU in the constraints also increases the complexity of solving the optimization problem. The objectives in Problem 1 affect the DL transmit power of the FD BS and the receiving signals of DT, while the objectives in Problem 2 affect the UL transmit power of the UL CU and the receiving signals of the DR. Further discussion is proposed in the next section.

## 3. Solution of the Optimization Problem

In this part, we first solve the two sub-problems with the Pareto optimality. Then, we analyze the network computational complexity. 

Assume that for minimizing total energy consumption, the transmit power of the UL CU first decreases which impairs the objects in Problem 1. The transmit power objects of Problem 1, ${\left\|w_{DT}\right\|}^{2}$ and Tr(Z), which result in significant SI should decrease to satisfy the SINR requirement $\Gamma_{req}^{UL}$. Besides, as for the links between DUs, the low power of AN causes a higher risk on the information leakage to the RU. Thus, the DT lowers the transmit power to DRs to meet the security requirement $C_{DR-E}$. It seems to converge to a reasonable circumstance that each of the allocated user powers approximately achieves the minimum SINR requirements. However, in practice, the user QoS is dynamically changing all the time. For example, $\Gamma_{req}^{DT}$ increases when DT needs a higher bandwidth for a live high-quality video. A higher $\Gamma_{req}^{DT}$ directs to a higher ${\left\|w_{DT}\right\|}^{2}$, which puts higher SI on the UL channel. This means $P_{UL}$ also needs to increase to compensate this interference for satisfying the minimum required QoS $\Gamma_{req}^{UL}$. As for security, the increment in the BS emission power also causes a higher risk on the information leakage which leads to another power increment of AN. This further impacts the RUs as they need more power to ensure QoS. Therefore, the objectives in the two sub-problems conflict with each other. When we pursue an object to minimize the system consumed power, the result tends to become higher. 

To overcome the shortcomings, the multi-objective optimization (MOO) problem is adopted with the concept of Pareto optimality. We first denote sub-problem i as $Q_{i}\left(Z,w_{DT},w_{DR1},w_{DR2},P_{UL}\right)$. A resource allocation policy that can be set as $\left\{Z,w_{DT},w_{DR1},w_{DR2},P_{UL}\right\}$ can achieve the Pareto optimal if and only if all the set members satisfy $Q_{i}\left(Z,w_{DT},w_{DR1},w_{DR2},P_{UL}\right)\geq Q_{i}^{*},\forall i\in \left\{1,2\right\},$ where $Q_{i}^{*}$ represents the optimal objective value of Problem i. To capture the complete Pareto optimal set, we resort to the weighted Tchebycheff scheme that can jointly solve the trade-off between the two sub-problems in MOO as shown in Problem 3 [10].

Problem 3:

(29) $$\begin{array}{l} \underset{Z,w_{DT},w_{DR1},w_{DR2},P_{UL},\tau }{\mathrm{minimize}}\tau ,\\ \;s.t.C1-C10,\\ \;\;C11:\lambda_{i}\left(Q_{i}-Q_{i}^{*}\right)\leq \tau ,\forall i\in \left\{1,2\right\}, \end{array}$$

where

(30) $$Q_{1}\left(Z,w_{DT},w_{DR1},w_{DR2},P_{UL}\right)={\left\|w_{DT}\right\|}^{2}+\mathrm{Tr}\left(Z\right)$$

and

(31) $$Q_{2}\left(Z,w_{DT},w_{DR1},w_{DR2},P_{UL}\right)={\left\|w_{DR1}\right\|}^{2}+{\left\|w_{DR2}\right\|}^{2}+P_{UL}$$

τ denotes an auxiliary optimization variable that targets the total transmit power minimization of the trusted UL and DL users. Variable $\lambda_{i}\geq 0,\sum_{i}\lambda_{i}=1,$ specifies the priority of the *i*-th objective compared to the other objectives and reflects the preference of the system operator [15].

As C4–C6 are non-convex constraints and C4–C7 have uncertain CSI, we consider using a linear relaxation approach to transform C4–C6 into a set of linear matrix inequalities (LMI) and the generalized S-procedure approach to solve the infinite number of inequality constraints C4–C7 produced by CSI uncertainty.

First, to handle the non-convex constraints C4–C6, we note the implications in the following equivalent transformation:

(32) $$C4\Leftrightarrow \tilde{C}4:P_{UL}e_{E}e_{E}^{H}⪯\xi_{tol}^{CU}X_{E}^{},\;e_{E}\in \Omega_{e},L_{E}\in \Omega_{E},$$

(33) $$C5\Leftrightarrow \tilde{C}5:L_{E}^{H}w_{DT}w_{DT}^{H}L_{E}^{}⪯\xi_{tol}^{DT}X_{E}^{-1},\;L_{E}\in \Omega_{E},$$

and

(34) $$C6\Leftrightarrow \tilde{C}6:L_{DT}^{H}\left(w_{DR1}^{}w_{DR1}^{H}+w_{DR2}^{}w_{DR2}^{H}\right)L_{DT}⪯\xi_{tol}^{DR}X_{E}^{},\;L_{DT}^{}\in \Omega_{DT},L_{E}\in \Omega_{E},$$

where: $R_{tol}^{CU}=\log_{2}\left(1+\xi_{tol}^{CU}\right)$, $R_{tol}^{DT}=\log_{2}\left(1+\xi_{tol}^{DT}\right)$, and $R_{tol}^{DR}=\log_{2}\left(1+\xi_{tol}^{DR}\right)$.

Next, note that $\tilde{C}4$–$\tilde{C}6$ and C7 still involve an infinite number of inequality constraints [17], and we consider introducing the generalized S-procedure to solve it.

Let $f\left(X\right)=X^{H}AX+X^{H}B+B^{H}X+C$, and D⪰0. For t≥0, f(X)⪰0, $\forall X\in \left\{X|\mathrm{Tr}\left(DXX^{H}\right)\leq 1\right\}$ is equivalent to

(35) $$\left[\begin{array}{cc} C & B^{H}\\ B & A \end{array}\right]-t\left[\begin{array}{cc} I & 0\\ 0 & -D \end{array}\right]⪰0.$$

Since $\tilde{C}4$ involves two coupled estimation error variables, a slack matrix variable $M_{CU}\in$ **ℋ***^M^* first needs to be introduced to handle it. In particular, $\tilde{C}4$ is equivalently represented by

(36) $$\tilde{C}4a:P_{UL}e_{E}e_{E}^{H}⪯M_{CU},e_{E}\in \Omega_{e},$$

(37) $$\tilde{C}4b:M_{CU}⪯\left(\xi_{tol}^{CU}-1\right)X_{E}^{},L_{E}\in \Omega_{E}.$$

Then, we apply (35) to $\tilde{C}4a$ and $\tilde{C}4b$ to get $\bar{C}4a$ and $\bar{C}4b$.

(38) $$\bar{C}4a:R_{\bar{C}4a}\left(M_{CU},P_{UL},\alpha_{CU}\right)\;\begin{array}{l} =[\begin{array}{cc} -P_{UL}{\hat{e}}_{E}{\hat{e}}_{E}^{H}+M_{CU}-\alpha_{CU}I_{N} & -P_{UL}{\hat{e}}_{E}\\ -P_{UL}{\hat{e}}_{E}^{H} & -P_{UL}+\alpha_{CU}\varepsilon_{e}^{-2} \end{array}] \end{array}⪰0$$

for $\alpha_{CU}\geq 0$, and

(39) $$\bar{C}4b:R_{\bar{C}4b}\left(Z,M_{CU},\beta_{CU}\right)=\;\left[\begin{array}{cc} \xi_{tol}^{CU}{\hat{L}}_{E}^{H}Z{\hat{L}}_{E}^{}+\left(\xi_{tol}^{CU}\sigma^{2}-\beta_{CU}\right)I_{N}-M_{CU} & \xi_{tol}^{CU}{\hat{L}}_{E}^{H}Z\\ \xi_{tol}^{CU}Z{\hat{L}}_{E}^{} & \xi_{tol}^{CU}Z+\beta_{CU}\varepsilon_{e}^{-2}I_{M} \end{array}\right]⪰0$$

for $\beta_{CU}\geq 0$.

By substituting $L_{E}={\hat{L}}_{E}+\Delta L_{E}$ into (33), we have

(40) $$\Delta L_{E}^{H}\left(\xi_{tol}^{DT}Z-W_{DT}\right)\Delta L_{E}^{}+\Delta L_{E}^{H}\left(\xi_{tol}^{DT}Z-W_{DT}\right){\hat{L}}_{E}^{}\;+L_{E}^{H}\left(\xi_{tol}^{DT}Z-W_{DT}\right)\Delta L_{E}^{}+{\hat{L}}_{E}^{H}\left(\xi_{tol}^{DT}Z-W_{DT}\right){\hat{L}}_{E}^{}\;+\xi_{tol}^{DT}\sigma^{2}I_{N}⪰0$$

where $\Delta L_{E}\in \left\{\Delta L_{E}|\mathrm{Tr}\left(\varepsilon_{E}^{-2}\Delta L_{E}\Delta L_{E}^{H}\right)\leq 1\right\}$ and $W_{DT}=w_{DT}w_{DT}^{H}$. Then, constraint $\tilde{C}5$ is equivalently written as

(41) $$\bar{C}5:R_{\bar{C}5}\left(W_{DT},Z,t\right)\;=\left[\begin{array}{cc} \xi_{tol}^{DT}{\hat{L}}_{E}^{H}Z{\hat{L}}_{E}^{}+\left(\xi_{tol}^{DT}\sigma^{2}-t\right)I_{N} & \xi_{tol}^{DT}{\hat{L}}_{E}^{H}Z\\ \xi_{tol}^{DT}Z{\hat{L}}_{E}^{} & \xi_{tol}^{DT}Z+t\varepsilon_{E}^{-2}I_{M} \end{array}\right]\;-B_{L_{E}}^{H}W_{DT}B_{L_{E}}⪰0$$

where $B_{L_{E}}=\left[\begin{array}{cc} {\hat{L}}_{E} & I_{M} \end{array}\right]$.

Similar to $\tilde{C}4,$ $\tilde{C}6$ is equivalently represented by

(42) $$\tilde{C}6a:L_{DT}^{H}W_{DR}L_{DT}^{}⪯M_{DR},L_{DT}^{}\in \Omega_{DT}$$

(43) $$\tilde{C}6b:M_{DR}⪯\left(\xi_{tol}^{DR}-1\right)X_{E}^{},L_{E}\in \Omega_{E},$$

where $M_{DR}\in$ **ℋ***^N^* and $W_{DR}=w_{DR1}^{}w_{DR1}^{H}+w_{DR2}^{}w_{DR2}^{H}$.

Then, we apply (35) to $\tilde{C}6a$ and $\tilde{C}6b$ to get $\bar{C}6a$ and $\bar{C}6b$.

$$\bar{C}6a:R_{\bar{C}6a}\left(M_{DR},W_{DR},\alpha_{DR}\right)$$

(44) $$\left[\begin{array}{cc} -{\hat{L}}_{DT}^{H}W_{DR}{\hat{L}}_{DT}^{}+M_{DR}-\alpha_{DR}I_{N} & -W_{DR}{\hat{L}}_{DT}^{}\\ -{\hat{L}}_{DT}^{H}W_{DR} & -W_{DR}+\alpha_{DR}\varepsilon_{DT}^{-2}I_{N} \end{array}\right]⪰0$$

for $\alpha_{DR}\geq 0$, and

(45) $$\bar{C}6b:R_{\bar{C}6b}\left(Z,M_{DR},\beta_{DR}\right)=\left[\begin{array}{cc} \xi_{tol}^{DR}{\hat{L}}_{E}^{H}Z{\hat{L}}_{E}^{}+\left(\xi_{tol}^{DR}\sigma^{2}-\beta_{DR}\right)I_{N}-M_{DR} & \xi_{tol}^{DR}{\hat{L}}_{E}^{H}Z\\ \xi_{tol}^{DR}Z{\hat{L}}_{E}^{} & \xi_{tol}^{DR}Z+\beta_{DR}\varepsilon_{DT}^{-2}I_{M} \end{array}\right]⪰0$$

for $\beta_{DR}\geq 0$. Similarly, constraint C7 is equivalently written as

(46) $$\bar{C}7:R_{\bar{C}7}\left(W_{DT},Z,t_{RU}\right)=\left[\begin{array}{cc} {\hat{L}}_{E}^{H}Z{\hat{L}}_{E}^{}-t_{RU}\varepsilon_{E}^{-2}I_{N}-\frac{E_{RU}^{}}{\mu } & {\hat{L}}_{E}^{H}Z\\ Z{\hat{L}}_{E}^{} & Z+t_{RU}I_{M} \end{array}\right]-B_{L_{E}}^{H}W_{DT}B_{L_{E}}⪰0$$

for $t_{RU}\geq 0$.

Now, the MOO problem (29) becomes a convex semidefinite programming (SDP) and can be written as

(47) $$\begin{array}{l} \underset{Z,w_{DT},w_{DR1},w_{DR2},P_{UL},\tau }{\mathrm{minimize}}\tau ,\\ \;s.t.C1-C3,C8-C11, \end{array}\;\bar{C}4a:R_{\bar{C}4a}\left(M_{CU},P_{UL},\alpha_{CU}\right)⪰0\;\bar{C}4b:R_{\bar{C}4b}\left(Z,M_{CU},\beta_{CU}\right)⪰0\;\bar{C}5:R_{\bar{C}5}\left(W_{DT},Z,t\right)⪰0\;\bar{C}6a:R_{\bar{C}6a}\left(M_{DR},W_{DR},\alpha_{DR}\right)⪰0\;\bar{C}6b:R_{\bar{C}6b}\left(Z,M_{DR},\beta_{DR}\right)⪰0\;\bar{C}7:R_{\bar{C}7}\left(W_{DT},Z,t_{RU}\right)⪰0.$$

The convex problem (47) is efficiently optimized by the standard convex programming software named CVX [18]. Note that once we find a rank-one matrix to be the solution of the relaxed SDP problem, the matrix can also be used as a solution of the original problem of (47). Next, we proof the existence of an optimal solution of (47).

**Proof.**  The proof of the existence of an optimal solution of (47) is given in the Appendix A. □

In the proposed secure FD-SWIPT network, the CSI received by a device is assumed to be bounded. This circumstance directs the infinite number of CSI values for a user to compute the accurate CSI of another one. Besides, considering the constraints, we treat the optimization problem as a non-convex problem. To directly apply the *S*-procedure method to the power minimization problem, we introduce several leverage parameters into the non-convex constraints with bounded CSI values. Then, the constraints can be written as equivalent matrices with linear form elements. At last, the non-convex problem is transformed into a convex form, which can be classified as the relaxed SDP problem.

## 4. Simulation Results

The performance of the proposed MOO resource allocation algorithm is investigated in this section. Table 2 specifies the most important simulation parameters. The RU is equipped with *N* = 2 antennas. The cell BS is on the center of the cellular with *M* = 3 antennas with a radius of 1000 m. The small-scale fading is modeled by Rayleith distribution and the large-scale fading can be calculated by (*d*/*d_0_*)^−^^*η*^, where *d* is the distance between two network devices and *d_0_* is the reference distance, which is set to be 100 m. Besides, the path loss exponent *η* is set to be 3. We set the communication radius of the D2D group as 300 m.

### 4.1. Convergence of the Proposed Algorithm

**Figure 2:**
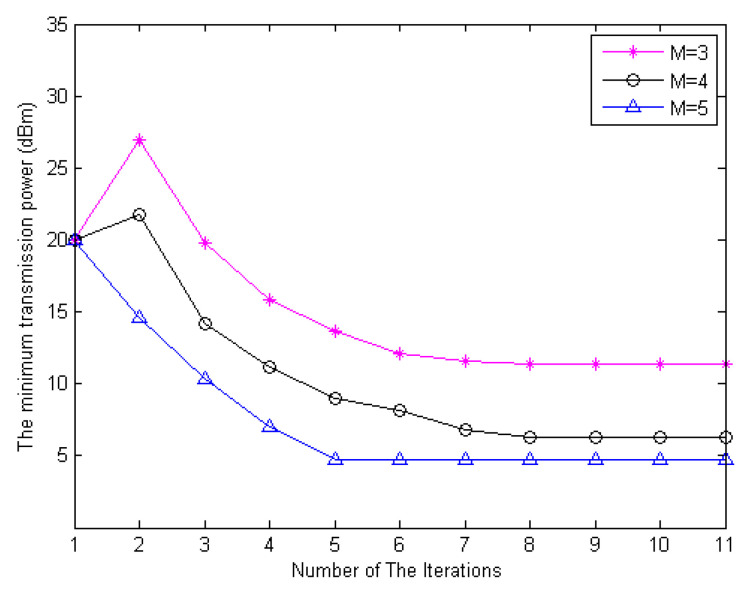
The minimum transmit power versus the number of iterations for the proposed algorithm with an iteration interval of $\bar{\Delta }=0.01$.

The minimum transmit power of the users versus the number of iterations is shown in Figure 2. We set the initial transmit power of the network as 20 dBm. The results in Figure 2 are averaged over 1000 independent adaptation processes which have different locations of the users in the cellular. It can be observed that, with an iteration interval of $\bar{\Delta }=0.01,$ the proposed algorithm has a fast convergence rate within 10 iterations. It is shown in Figure 2 that the proposed power minimization approach is capable of saving more power when the number of antennas on the FD BS become larger. This is because the inherent spatial diversity comes up with more antennas which creates a benefit for power efficiency. With that, the system can indeed combat the path loss with a smaller portion of radiated power.

**Figure 3:**
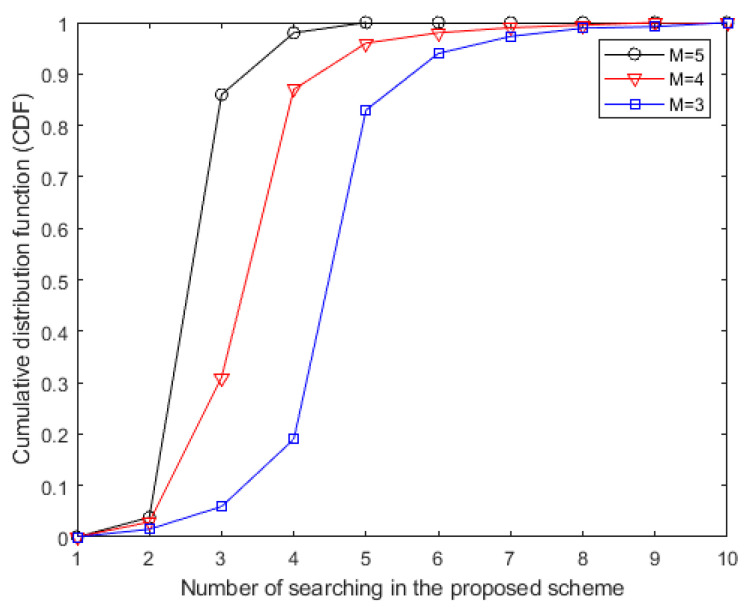
The cumulative distribution function (CDF) of the proposed minimum transmission power allocation scheme with imperfect channel state information (CSI) under the parameters of the FD BS antenna numbers with *M* = 5, *M* = 4 and *M* = 3.

In Figure 3, the cumulative distribution function (CDF) of the proposed algorithm is displayed using the bounded CSI error model to deal with the imperfect CSI. It can be observed that, with more antennas at the FD BS, the proposed algorithm has a faster convergence rate within 10 iterations because of the full accomplishment of the available degrees of freedom (DoF) in globally optimal resource allocation. Summarizing the results obtained from Figure 2 and Figure 3, we can observe the convergence of the power minimization algorithm with the same parameters.

### 4.2. Transmit PowerTrade-off Region

**Figure 4:**
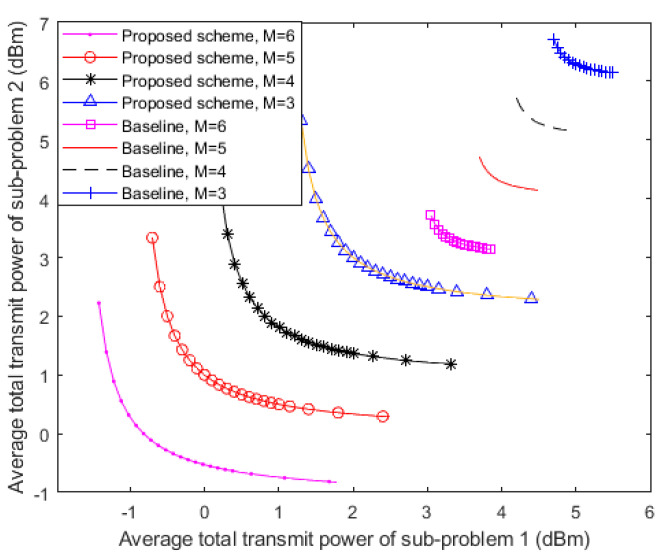
Average transmit power trade-off region between the two sub-problems of each device achieved by the proposed power minimization resource allocation policy. The baseline is simulated under the parameters of the FD BS antenna numbers with *M* = 6, *M* = 5, *M* = 4 and *M* = 3.

The trade-off between the average transmit power of each device in sub-problem 1 and sub-problem 2 with imperfect CSI and different antenna numbers on the FD BS is shown in Figure 4. It can be observed that the average transmit power consumption of each user in sub-problem 1 is monotonically decreased with respect to the higher average transmit power of the users in sub-problem 2, which means that maximizing the power consumption of one sub-problem leads to lower power consumption of the other and vice versa. In other words, the results in Figure 4 confirm that minimizing the transmit power of UL and DL in FD devices creates conflicting design objectives. For instance, when *M* = 4, 3 dBm transmit power of sub-problem 2 can be saved by a 3.5 dBm increment in the total transmit power of sub-problem 1. In addition, it can be indicated in the figure that a significant amount of total transmit power is saved with the increment in the number of FD BS antennas. This is because additional antennas offer extra (DoF) to facilitate more power efficiency. Under this circumstance, the FD BS does not need to transmit extra power for neutralizing the increasing potential of information leakage anymore. With advanced interference management technologies in the next-generation wireless network, the increment in the FD BS antennas number has a good prospect in providing higher power efficiency for the wireless communication system.

Considering the baseline scheme, ZF-BF is adopted as the power emission approach for comparison. It is shown in Figure 4 that the resource allocation approach in this paper is capable of saving more power because the curves are below the trade-off regions from the baseline scheme. It can also be indicated from the depiction that when *M* = 4, only 0.6 dBm power from sub-problem 2 can be saved by around a 1.5 dBm increment in the power from sub-problem 1. In fact, because the baseline scheme cannot exploit the full benefits of having available DoF from more antennas, the proposed approach is more energy-efficient.

### 4.3. Average Power Consumption versus Transmit SNR

**Figure 5:**
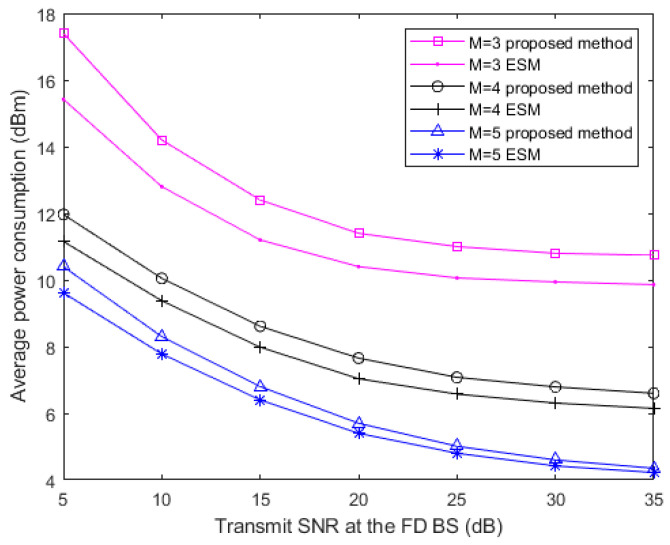
Average transmit power of the users versus the transmit SNR of the FD BS for different total antenna numbers at the FD BS with *M* = 5, *M* = 4 and *M* = 3, in two methods.

Figure 5 depicts the relationships between average power consumption of the users in the FD system and the transmit signal to noise ratio (SNR) $\rho_{BS}$ for different antenna numbers at the FD BS, here $\rho_{BS}={\left\|w_{DT}\right\|}^{2}/\sigma^{2}$. From Figure 5, it is observed that the average power consumption of the users is getting smaller while $\rho_{BS}$ at the FD BS keeps getting bigger when M increases. In terms of *M*, more antennas create the condition that the direction of the beamforming vector can be more accurately steered towards the users. This situation reduces the power consumption and the leakage of the AN power in the system. As we can also see from Figure 5, the average power consumption decreases gradually with the transmit SNR at the FD BS. However, the transmission power cannot be infinitely small because of the existence of constraints for guaranteeing the EH task of the RU. Furthermore, we compare the performance between two methods: the exhausted search method (ESA) with perfect CSI and our proposed method with the same parameters. The ESA can give optimal search results after infinite times of iterations but costs a lot of time. Within a lower computing resource cost, the results of our proposed method indicate that multiple antennas (*M* > 3) in this network can effectively improve the performance of the proposed algorithm to find the optimal solution. In fact, more antennas provide a more stringent QoS in this situation, and this reduces the average power consumption of SWIPT and interference management.

### 4.4. The Minimum Transmit Power in Different Situations

**Figure 6:**
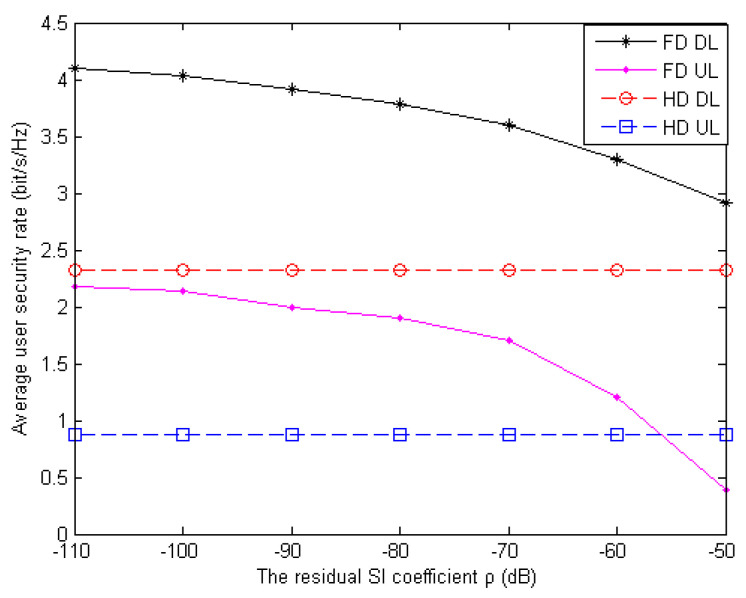
Average user security rate versus the self-interference (SI) coefficient of the antennas for the downlink (DL) and uplink (UL) in different FD and HD schemes.

In Figure 6, the impact of the residual SI ρ of each FD antenna on the achievable average DL and UL security rate of the users is presented with bounded CSI. It can be observed from the figure that the security rate performance in FD communication degrades as ρ increases, while that in the HD situation remains the same. Moreover, the UL security rate is always lower than the DL security rate under the same ρ. This is because, as compared with the UL, RU exerts a negligible impact on the DL due to the strong interference cancellation ability of the FD BS along with the DT. Nevertheless, with more SI, the FD BS needs to transmit higher power to satisfy the network data rate which brings information leakage to the RU. Thus, the proposed scheme has to be devoted to allocate more power to the AN injection for wireless transmission secrecy. When the total energy of the system is limited, the UL network security data rate of FD becomes lower than HD.

In the above simulations, we make efforts on minimizing the network transmit power while jointly guaranteeing the QoS and the secrecy limits. However, the transmission power cannot become infinitely small because of another existence of constraints for guaranteeing the EH task of the RU. In this paragraph, we pay attention to satisfying the EH constraint with perfect and imperfect CSI under NOMA and OMA, respectively.

**Figure 7:**
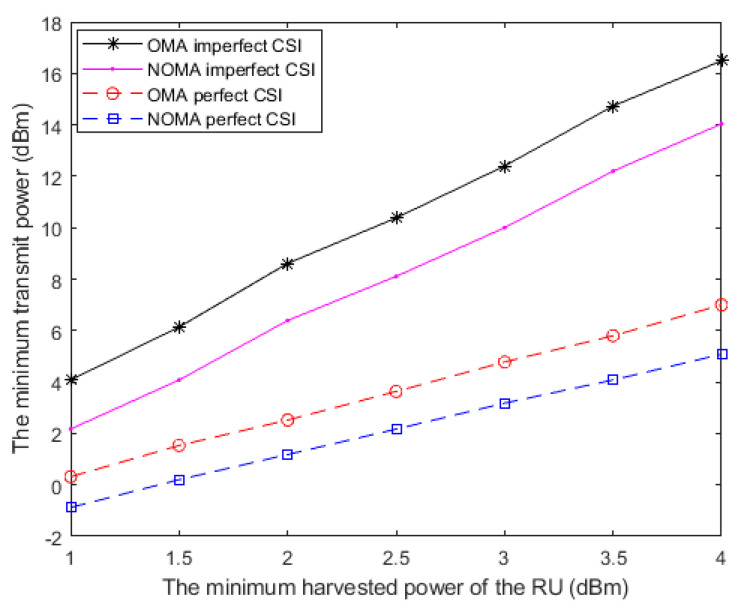
The minimum transmit power versus the minimum harvested power of the roaming users (RU) in the system with perfect and imperfect CSI.

Figure 7 depicts the minimum transmit power versus the minimum EH value of the RU in the system with perfect and imperfect CSI. It can be seen from the result by the proposed algorithm that the transmit power consumption by adopting NOMA is lower than that of adopting OMA in the scenarios of perfect and imperfect CSI. As a matter of fact, NOMA outperforms OMA in the network secrecy rate. This situation leads to a relatively low power requirement for guaranteeing the network secrecy rate and the energy consumption of AN. It is also seen that the scenario with imperfect CSI causes a significant impact on the minimum transmit power because of the estimation errors. Moreover, as the EH requirement of RU increases, a higher level of the minimum transmit power is needed to fulfill the secrecy of the information transfer and the QoS requirement for the EH task.

## 5. Conclusions

In this paper, a novel secure FD SWIPT scheme was proposed using NOMA and D2D, where a practical bounded CSI estimation method was applied. The secrecy rate was defined to guarantee the secure transmission and a linear EH model was built to evaluate the energy saving of the system. The proposed network transmit power minimization problem was divided into two sub-problems and formulated as an MOO problem via the weighted Tchebycheff approach. A method with LMI was used to transform the non-convex constraints into convex form. Taking into account the potential eavesdropper RU with imperfect CSI for the robust power-efficient resource allocation, a bounded transmission beamforming vector design along with an AN vector was used. Through this method, the system could satisfy the requirements of the secrecy rates as well as the EH task. Numerical simulation results not only validated the convergence performance but also showed a trade-off between the UL and DL transmit power of the MOO algorithm. Furthermore, we noted that by FD and NOMA, the utility of the proposed iterative algorithm is higher than that with HD and OMA with the same parameter. Moreover, our proposed SWIPT optimization scheme is efficient to satisfy the EH requirement.

## Figures and Tables

**Table 1:** Abbreviation index.

Full Names	Abbreviations
full-duplex	FD
half-duplex	HD
simultaneous wireless information and power transfer	SWIPT
non-orthogonal multiple access	NOMA
orthogonal multiple access	OMA
device-to-device	D2D
channel state information	CSI
multi-objective optimization	MOO
linear matrix inequality	LMI
artificial noise	AN
energy harvesting	EH
uplink	UL
downlink	DL
quality of service	QoS
spectrum efficiency	SE
co-channel interference	CCI
self-interference	SI
semidefinite programming	SDP
positive semidefinite	PSD
D2D transmitter	DT
D2D receivers	DR
D2D user	DU
cellular users	CU
roaming user	RU
signal-to-interference-plus-noise ratio	SINR
signal to noise ratio	SNR
additive white Gaussian noise	AWGN
zero-force beamforming	ZF-BF
minimum mean square error beamforming	MMSE-BF
cumulative distribution function	CDF

**Table 2:** System parameters.

Parameters	Value
Carrier center frequency and system bandwidth	2.3 GHz and 200 kHz
Maximum estimation error	5%
Path loss exponent and SI cancellation constant, *ρ*	3.3 and −90 dB [19]
DL and UL user noise power σ^2^	−113 dBm
Maximum tolerable data rate at RU for CU	1 bit/s/Hz
Maximum tolerable data rate at RU for BS	1 bit/s/Hz
Maximum tolerable data rate at RU for DT	1 bit/s/Hz
Minimum required SINR for CU	3 dB
Minimum required SINR for BS	8 dB
Minimum required SINR for DT	10 dB

## Appendix A

The Lagrange function of problem (47) is as follows (take $w_{DT}$ as an example):

(A1) $$\begin{array}{ll} ℒ≜ & {\left\|W_{DT}\right\|}^{2}-\mathrm{tr}\left(\Phi_{DT}{\bar{R}}_{\bar{C}5}\left(W_{DT},Z,t\right)\right)\\ & -\mathrm{tr}\left(\Phi_{E}{\bar{R}}_{\bar{C}7}\left(W_{DT},Z,t_{RU}\right)\right)-A_{DT}W_{DT} \end{array}$$

where $\Phi_{DT}$ and $\Phi_{E}$ are dual variables, and $A_{DT}$ is the adjoint variable of $W_{DT}$.

(A2) $${\bar{R}}_{\bar{C}5}\left(W_{DT},Z,t\right)=\Lambda_{DT}+B_{L_{E}}^{H}\left(W_{DT}-\xi_{tol}^{DT}Z\right)B_{L_{E}}$$

(A3) $${\bar{R}}_{\bar{C}7}\left(W_{DT},Z,t_{RU}\right)=\Lambda_{E}-B_{L_{E}}^{H}\left(Z-W_{DT}\right)B_{L_{E}}$$

where $\Lambda_{DT}=\left[\begin{array}{cc} -\left(\xi_{tol}^{DT}\sigma^{2}-t\right)I_{N} & 0\\ 0 & -t\varepsilon_{E}^{-2}I_{M} \end{array}\right]$ and $\Lambda_{E}=\left[\begin{array}{cc} t_{RU}\varepsilon_{E}^{-2}I_{N}+\frac{E_{RU}^{}}{\mu } & 0\\ 0 & -t_{RU}I_{M} \end{array}\right]$. With Karush–Kuhn–Tucker (KKT) conditions, we have

(A4) $$\nabla_{W_{DT}}L=0$$

(A5) $$\Phi_{DT}R_{\bar{C}5}\left(W_{DT},Z,t\right)=0$$

(A6) $$\Phi_{E}R_{\bar{C}7}\left(W_{DT},Z,t_{RU}\right)=0$$

(A7) $$A_{DT}W_{DT}=0$$

(A8) $$A_{DT}⪰0,\Phi_{DT}⪰0,\Phi_{E}⪰0.$$

where $A_{DT}=I_{N}-B_{L_{E}}\left(\Phi_{DT}-\Phi_{E}\right)B_{L_{E}}^{H}$. Further, we define that $G≜I_{N}+B_{L_{E}}\Phi_{E}B_{L_{E}}^{H}$ and $r_{G}=\mathrm{rank}\left(G\right)$ Thus,

(A9) $$A_{DT}≜G-B_{L_{E}}\Phi_{DT}B_{L_{E}}^{H}$$

Next, we proof $\mathrm{rank}\left(B_{L_{E}}\Phi_{DT}B_{L_{E}}^{H}\right)\leq 1$. Substituting part of (A5) with (A2), we have

(A10) $$\Lambda_{DT}\Phi_{DT}+B_{L_{E}}^{H}\left(W_{DT}-\xi_{tol}^{DT}Z\right)B_{L_{E}}\Phi_{DT}=0$$

By post-multiplying $B_{L_{E}}^{H}$, we get

(A11) $$\Lambda_{DT}\Phi_{DT}B_{L_{E}}^{H}+B_{L_{E}}^{H}\left(W_{DT}-\xi_{tol}^{DT}Z\right)B_{L_{E}}\Phi_{DT}B_{L_{E}}^{H}=0$$

Note that $[I_{M}0]B_{L_{E}}^{H}=I_{M}$ and define $\mu_{DT}=\xi_{tol}^{DT}\sigma^{2}-t$, we can get

(A12) $$\left[0\;I_{M}\right]\Lambda_{DT}=\mu_{DT}\left[I_{M}0\right]=\mu_{DT}\left(B_{L_{E}}-\left[L_{DT}0_{M}\right]\right)$$

After both sides of (A6) are multiplied by $[0\;I_{DT}]$, we can get

(A13) $$\begin{array}{l} \left(\mu_{DT}I_{M}+W_{DT}-\xi_{tol}^{DT}Z\right)B_{L_{E}}\Phi_{DT}B_{L_{E}}^{H}\\ =\mu_{DT}\left[L_{DT}0_{M}\right]\Phi_{DT}B_{L_{E}}^{H} \end{array}$$

**Lemma** **A1.** *If the Hermitian matrix $M=\left[\begin{array}{cc} M_{11} & M_{12}\\ M_{21} & M_{22} \end{array}\right]$⪰0, then it immediately follows that $M_{11}$ and $M_{22}$ must be the positive semi-definite (PSD) matrix [20]*.

Here, we can claim that $\mu_{DT}I_{M}+W_{DT}-\xi_{tol}^{DT}Z$⪰0 and the left side of this formula is nonsingular. We post-multiply a nonsingular matrix without changing the matrix rank and get

(A14) $$\begin{array}{l} \mathrm{rank}\left(B_{L_{E}}\Phi_{DT}B_{L_{E}}^{H}\right)\\ =\mathrm{rank}\left(\left(\mu_{DT}I_{M}+W_{DT}-\xi_{tol}^{DT}Z\right)B_{L_{E}}\Phi_{DT}B_{L_{E}}^{H}\right)\\ =\mathrm{rank}\left(\mu_{DT}\left[L_{DT}0_{M}\right]\Phi_{DT}B_{L_{E}}^{H}\right)\\ \leq \mathrm{rank}\left(\left[L_{DT}0_{M}\right]\right)\leq 1. \end{array}$$

**Lemma** **A2.**
*Let $X_{1}$ and $X_{2}$ be two matrixes of the same size. Then, it holds true that rank ($X_{1}$ − $X_{2}$) ≥ rank ($X_{1}$) - rank ($X_{2}$) [21].*

Through Lemma A2, we can derive $\mathrm{rank}\left(A_{DT}\right)\geq r_{G}-1$. If G is a positive definite, $r_{G}=M$ and $\mathrm{rank}\left(A_{DT}\right)\geq M-1$. However, if rank ($A_{DT}$) = M, i.e., $A_{DT}$ becomes a full-rank matrix. Following 54, we note that $W_{DT}=0$ cannot be the optimal solution to (47). Therefore, we have rank ($A_{DT}$) = M−1, and with 54, we can conclude rank ($W_{DT}$) = 1. Hence, $W_{DT}$ can be substituted by $\nu \varphi \varphi^{H}$, and here,φ spans the null space of $A_{DT}$ with ν>0.

The next step is to proof G⪰ **0**, which means to show that G is a positive definite matrix. Since we have the conditions that $A_{DT}$⪰ **0** and $B_{L_{E}}\Phi_{DT}B_{L_{E}}^{H}$⪰ **0** for $\Phi_{DT}$⪰ **0** and G⪰ **0**, by contradiction, we can explain that G⪰ **0** must always hold. Assume 0 is the minimum eigenvalue of G. Then, there exists at least a vector y≠0 such that $y^{H}Gy=0.$ According to (A4), it follows that $y^{H}A_{DT}y=y^{H}B_{L_{E}}\Phi_{DT}B_{L_{E}}^{H}y=-{\left|y^{H}B_{L_{E}}\Phi_{DT}^{1/2}\right|}^{2}$<0. This means that $A_{DT}$ is not PSD and violates the KKT conditions in (A7). Hence, we conclude that G⪰ **0** must hold.
